# Prevalence of Degenerative Spinal Hot Spots on Bone Scintigraphy Among Orthopedic Patients

**DOI:** 10.7759/cureus.77779

**Published:** 2025-01-21

**Authors:** Helena Milavec, Victoria Schimmelpenning, Nikki Rommers, Mara I Dimitriu, Martin Jaeger, Clément L Werner, Robin Brugger

**Affiliations:** 1 Orthopaedic Surgery and Traumatology, Aarau Cantonal Hospital, Aarau, CHE; 2 Orthopaedic Surgery and Traumatology, University Hospital Bern, University of Bern, Bern, CHE; 3 Clinical Research, University Hospital Basel, University of Basel, Basel, CHE; 4 Spine Surgery, Etzelclinic, Center for Minimally Invasive Surgery, Pfaeffikon, CHE

**Keywords:** bone scintigraphy, nuclear imaging, spinal diagnostic, spinal hot spots, spinal imaging

## Abstract

Purpose: Emerging nuclear imaging technologies are advancing the field of spinal diagnostics for degenerative changes. This study aimed to describe the prevalence of spinal hot spots in an orthopedic population undergoing bone scintigraphy (BS) for reasons unrelated to the spine and to assess the correlation of these hot spots with the presence of corresponding spinal complaints.

Methods: A retrospective analysis of patients from the Department of Orthopedic Surgery and Traumatology who provided general consent for the use of their medical data. Adult patients who underwent single photon emission computed tomography (SPECT) over a two-year period for non-spinal complaints, along with a corresponding whole-body BS, were included. The primary endpoint was the prevalence of degenerative spinal hot spots detectable on BS. The secondary endpoint evaluated clinical complaints recorded in medical records for each spinal region.

Results: Among 30 patients (mean age: 66.1 years; 30% male, 70% female), the prevalence of spinal hot spots on BS was 23.3% (n=7) in the cervical spine, 10% (n=3) in the thoracic spine, 33.3% (n=10) in the lumbar spine, and 6.7% (n=2) in the sacroiliac joint (SIJ). Most patients with cervical and lumbar hot spots had corresponding spinal symptoms, such as pain, tenderness, or stiffness, documented in their medical records, whereas thoracic complaints were less commonly noted.

Conclusion: The study outlines the prevalence of spinal hot spots in various spinal regions among a general orthopedic population undergoing bone scans for non-spinal complaints. A strong correlation was found between spinal complaints and high uptake in the cervical spine and lower back. These findings highlight the added value of comprehensive bone scan evaluations in clinical practice.

## Introduction

Emerging nuclear imaging technologies are increasingly being utilized in spinal diagnostics for degenerative changes. Conventional imaging methods, such as computed tomography (CT) and magnetic resonance imaging (MRI), often have limited predictive value in identifying the root cause of back pain because the relationship between clinical symptoms and imaging findings is complex and inconsistent. Nuclear imaging techniques, such as bone scintigraphy (BS) or three-dimensional hybrid imaging combining single photon emission computed tomography (SPECT) and CT, expand diagnostic capabilities by enhancing the detection of osteoblastic remodeling and synovial changes, potentially identifying pain generators. SPECT/CT, in particular, provides higher spatial resolution and allows simultaneous assessment of bone metabolism and anatomical structures, which can aid in better identifying pathological areas and improving diagnostic accuracy [1-5].

Nuclear imaging techniques involve the administration of a radiotracer to evaluate bone metabolism and detect skeletal abnormalities, often referred to as "hot spots" or areas of high uptake. BS is typically performed as a whole-body scan for comprehensive assessment, and SPECT/CT is then used to provide more detailed anatomical and functional information about a specific region of interest [6]. The clinical relevance of spinal hot spots has been the subject of several recent studies [7-9]. However, despite the high diagnostic value of BS, comprehensive data on the prevalence and clinical implications of spinal hot spots in patients with non-spinal concerns, reflecting the general population, are still lacking.

This study aimed to assess the prevalence of spinal hot spots in patients undergoing BS and SPECT/CT for non-spinal concerns, reflecting a general population. Additionally, we evaluated whether symptoms related to the spine, such as pain, stiffness, or restricted mobility, were documented in the patient's medical records and assessed the predictive value of these findings.

## Materials and methods

Study design

Retrospective analysis of all available data from medical records and imaging reports of patients treated at the Department of Orthopedic Surgery and Traumatology of our tertiary institution. The study included only patients who provided general consent for the use of their medical data.

Population and analysis sets

The study population consisted of patients aged 18 and older who visited the general orthopedic outpatient department and underwent SPECT/CT for non-spinal pathologies (e.g., knee, hip, or ankle complaints) over a two-year period. We reviewed records of patients who had whole-body BS available. Cases were excluded if malignancy or infections were present, if the patient was under 18, or if general consent was not obtained. We analyzed the relationship between asymptomatic patients (those with no spinal complaints) and symptomatic patients (those with spinal complaints) in relation to the presence or absence of hot spots on BS. 

Data for spinal complaints were binary, indicating whether complaints were recorded for a particular spinal region, either in the past or present.

Imaging

Technetium-99m hydroxydiphosphonate BS were performed. The Images were reviewed by two senior nuclear medicine consultants who were responsible for the nuclear imaging report.

Analysis of images and medical records

We assessed the nuclear medicine reports for spinal hot spots (yes/no) across the cervical, thoracic, and lumbar spine, as well as the sacroiliac joint (SIJ). Additionally, we analyzed medical histories for any complaints or diagnoses related to the respective spinal regions. We summarized symptoms in the lower back and SIJ region as lower back pain (LBP).

Statistical analyses

Summary statistics of patient characteristics were presented for all patients, using frequencies and percentages for categorical variables, and either the mean and standard deviation or the median and interquartile range for numerical variables, depending on the distribution. This study was descriptive in nature, with no inferences drawn.

As a primary analysis, we assessed the prevalence of hot spots on BS for the entire spine, as well as the SIJ, and the lumbar, thoracic, and cervical spine separately. We provided an estimate of the prevalence along with the corresponding 95% Wilson confidence interval.

As a secondary analysis, we evaluated whether these patients had any mention of spinal issues (past or present) in their medical records and presented the proportion with the corresponding 95% Wilson confidence interval. 

As a tertiary analysis, we calculated positive and negative predictive values (PPVs and NPVs) to assess the diagnostic utility of BS. PPVs were defined as the proportion of patients with hot spots who had corresponding symptoms, while NPVs were defined as the proportion of patients without hot spots who were asymptomatic. Wilson confidence intervals for these predictive values were calculated to provide an estimate of variability.

Ethical approval

The protocol of this study was approved by the local ethics committee. Prior to commencement, written consent was obtained from all participating patients to use their medical data. The study was conducted in accordance with the Declaration of Helsinki and good clinical practice.

## Results

Patient characteristics

Our study population consisted of 30 patients, with a mean age of 66.1 years (age range: 39 to 83 years). The majority of patients were female (70% female, n=21; 30% male, n=9). Most patients presented with lower limb complaints, such as pain in the foot, hip, knee, or shoulder. A review of their medical records revealed that 13.3% (n=4) had undergone previous spinal surgery (thoracic, lumbar, or cervical) with metal implants, with all surgeries performed at least two years prior to imaging. Six (20%) reported cervical issues, one (3.3%) reported thoracic pain and nine (30%) had a history of LBP, including SIJ pain. The demographic characteristics of these patients are described in Table 1.

**Table 1:** Patient characteristics BS, bone scintigraphy; SPECT, single photon emission computed tomography; CT, computed tomography

Characteristic	Overall (n=30)
Age (years) (mean (SD))	65.1 (13.7)
Sex = male (%)	9 (30.0)
Indication for BS/SPECT/CT	n (%)
Foot	13 (43.3)
Hip	1 (3.3)
Knee	15 (50.0)
Shoulder	1 (3.3)
Previous back surgery = yes, with metal (%)	4 (13.3)
Cervical complaints in medical records = yes (%)	6 (20.0)
Thoracic complaints in medical records = yes (%)	1 (3.3)
Lower back complaints in medical records = yes (%)	9 (30.0)

Prevalence of hot spots in bone scintigraphy across different regions of the spine

**Figure 1:**
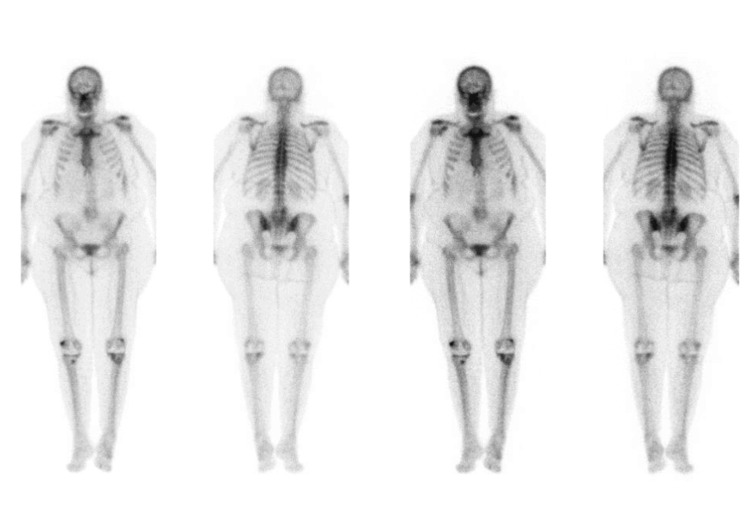
Example of spinal hot spots detectable on BS: facet joint arthritis L4-S1 on the right side BS, bone scintigraphy Image Credit: Authors

**Figure 2:**
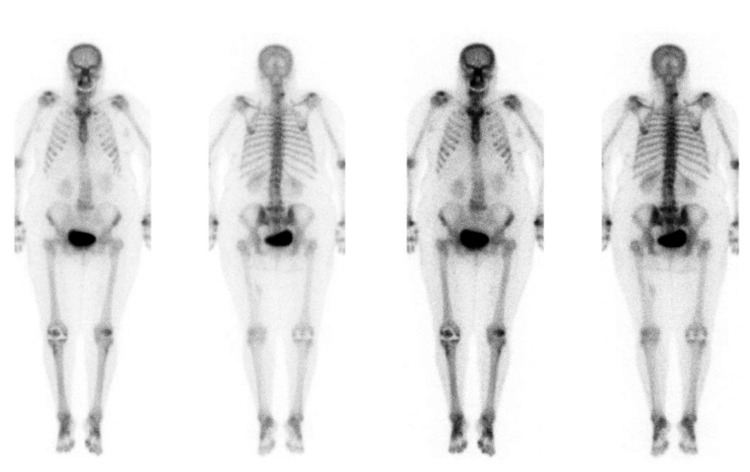
Example of spinal hot spots detectable on BS: facet joint arthritis in the low cervical spine on the right side BS, bone scintigraphy Image Credit: Authors

The prevalence of uptake on BS in each of the spinal regions is displayed in Table 2, along with the 95% CI. We found the highest prevalence in the lumbar spine with 33.3% (n=10), followed by the cervical spine with 23.3% (n=7). Spinal hot spots were rare in the thoracic spine with 10.0% (n=3), and even less in the SIJ with 6.7% (n=2). Notably, all patients with a high uptake in the SIJ also displayed a high uptake in the lumbar spine. Figure 1 and Figure 2 show examples of spinal hot spots detectable on BS. 

**Table 2:** Proportion of patients with uptake in each spinal region without spinal indication

Spinal region	n	Prevalence (95% CI) (%)
Cervical spine	7	23.30 (11.8, 40.9)
Thoracic spine	3	10.00 (3.5, 25.6)
Lumbar spine	10	33.30 (19.2, 51.2)
Sacroiliac joint	2	6.70 (1.8, 21.3)

Correlation of hot spots to symptoms

We observed variability in the correlation between spinal hot spots and symptoms across different spinal regions. In the analysis of the relationship between asymptomatic patients (those with no spinal complaints) and symptomatic patients (those with spinal complaints) in relation to the presence or absence of hot spots on BS. Among patients with hot spots, the following PPV were identified: 42.8% (3/7, 95% CI: 15.8 to 75%) in the cervical spine with neck pain, 33.3% (1/3, 95% CI: 1.7 to 79.2%) in the thoracic spine with thoracic pain, 40.0% (4/10, 95% CI 16.8 to 68.7%) in the lumbar spine with LBP, and 50.0% (1/2, 95% CI: 2.6 to 97.4%) in the SIJ with LBP (Table 3).

**Table 3:** Percentage of patients with symptoms correlating to hot spots BS, bone scintigraphy

Region	Hot spots/positive BS (n)	Correlating symptoms (%)	95% CI
Cervical spine	7	42.86% (3/7)	(15.8%, 75.0%)
Thoracic spine	3	33.33% (1/3)	(1.7%, 79.2%)
Lumbar spine	10	40.00% (4/10)	(16.8%, 68.7%)
Sacroiliac joint	2	50.00% (1/2)	(2.6%, 97.4%)

Patients without hot spots (negative BS) were more likely to be asymptomatic in the corresponding spinal region. Specifically, 87.0% (20/23, 95% CI: 67.9 to 95.5%) of patients without hot spots in the cervical spine were free of neck pain, while all patients (100%, 27/27, 95% CI: 87.5 to 100%) without hot spots in the thoracic spine were asymptomatic for thoracic complaints. Similarly, 75.0% (15/20, 95% CI: 53.1 to 88.8%) of patients without lumbar spine hot spots and 74.1% (20/27, 95% CI: 52.9 to 84.7%) of patients without SIJ hot spots were free of LBP (Table 4). 

**Table 4:** Percentage of patients without symptoms correlating to the absence of hot spots BS, bone scintigraphy

Region	No hot spots/negative BS (n)	Asymptomatic (%)	95% CI
Cervical spine	23	87.0% (20/23)	(67.9%, 95.5%)
Thoracic spine	27	100% (27/27)	(87.5%, 100%)
Lumbar spine	20	75.0% (15/20)	(53.1%, 88.8%)
Sacroiliac joint	27	74.1% (20/27)	(52.9%, 84.7%)

## Discussion

This is the first study to describe the prevalence of spinal hot spots detected on BS across different spinal regions in a general orthopedic population, reflecting findings in a non-specific group. Our results emphasize the added value of examining spinal findings in whole-body BS, which are readily available when performed alongside SPECT-CT, without incurring additional costs or radiation exposure. The use of additional tools available alongside the initial diagnostic process has proven useful in spinal imaging and other fields, where supplementary tools enhance diagnostic accuracy [10]. We observed a prevalence of high uptake ranging from 7% to 33.3% across different spinal regions, with the lumbar spine exhibiting the highest prevalence (33.3%, n=10), followed by the cervical spine (23.3%, n=7), the thoracic spine (10%, n=3), and the SIJ (6.7%, n=2).

The estimated annual prevalence reported in the literature for neck pain ranges from 4.8% to 79.5% (mean: 25.8%) [11], for thoracic back pain between 3.5% and 34.8% [12], and for low back pain (LBP), it ranges from 4.8% to over 50% [13]. In our study, the prevalence of spinal complaints, regardless of a positive or negative scan, ranged from 13% (n=4) found in the cervical spine to 30% (n=9) found in the lower back region, which is slightly lower than reported in the literature. To address suspected interobserver variability in clinical assessments of the SIJ, we combined LBP and SIJ pain into a single category referred to as pain in the lower back region.

Recent studies have focused on evaluating the diagnostic accuracy and clinical correlation of BS and SPECT/CT in patients with spinal complaints and the effectiveness of BS in the management of LBP [14-19]. However, its use as an appropriate imaging modality should be considered carefully given the increased radiation dose, especially in young individuals presenting with a benign disease. In our findings, the correlation between imaging findings and clinical symptoms underscores the potential clinical relevance of identifying spinal hot spots and highlights the supplementary information of BS in the clinical routine of orthopedic practice. However, some patients with negative BS also had reported spinal symptoms documented in their medical records, especially in the lumbar region. This highlights the complexity of interpreting incidental findings and underscores the need for comprehensive clinical assessment in patients undergoing imaging studies. However, the CIs are wide due to the limited number of patients, indicating considerable uncertainty in these estimates, and no formal conclusions can be drawn.

The highest PPV was observed in the cervical spine (42.9%), indicating that nearly half of the patients with hot spots in this region reported corresponding neck pain. However, the PPV for other regions, such as the thoracic and lumbar spine, was lower, underscoring the need for cautious interpretation. Conversely, the NPVs were generally high, particularly in the thoracic spine (100%) and cervical spine (87.0%), demonstrating that patients without hot spots are less likely to report symptoms in those regions. These findings emphasize the added value of incorporating predictive values into the interpretation of nuclear imaging results, offering a more nuanced understanding of the relationship between spinal hot spots and symptoms. The high NPVs demonstrate the diagnostic utility of BS in identifying asymptomatic patients, particularly in the thoracic and cervical regions, where negative scans are useful for excluding symptoms. However, the wide CIs, especially for PPVs, reflect the limited sample size, which precludes drawing formal conclusions. This highlights the need for larger studies to confirm the predictive value of BS in this context.

Limitations

Our study has several limitations that warrant consideration. The retrospective nature of the analysis limits our ability to establish causality and may be influenced by biases stemming from incomplete or inconsistent data collection, such as the lack of systematic documentation of spinal complaints. The absence of spinal complaints was not systematically queried or documented, potentially leading to an overestimation of NPVs. Mild or intermittent symptoms in patients without hot spots may have gone unreported, introducing bias toward higher NPVs. Furthermore, the lack of coherent findings in clinical assessments limited the interpretation of SIJ pain in comparison to LBP. Our findings are also limited by a small sample size, which affects the generalizability of our results and the precision of estimates, particularly for PPVs. We acknowledge the need for larger studies to validate our conclusions. The wide CIs observed in our analysis reflect this limitation, underscoring the need for larger studies to draw more robust conclusions regarding the association between spinal hot spots and clinical symptoms. While our study demonstrates a correlation between spinal hot spots and symptoms, the clinical significance of these findings, particularly in terms of their role in diagnosing or guiding treatment, remains an area for further exploration. We recognize that the potential utility of spinal hot spots as diagnostic markers or in treatment decision-making requires more in-depth investigation. Future studies should focus on validating these findings in larger, more diverse populations and exploring how spinal hot spots can be integrated into clinical practice to enhance diagnostic accuracy and improve patient outcomes.

## Conclusions

In conclusion, this study highlights the prevalence of spinal hot spots across various spinal regions in a general orthopedic population undergoing nuclear imaging for non-spinal complaints. BS is routinely performed alongside SPECT/CT and provides additional diagnostic insights by revealing areas of high uptake beyond the primary region of interest. The high NPVs observed underscore the utility of BS in identifying asymptomatic patients, while the moderate PPVs highlight the need for cautious interpretation of symptomatic correlations. These findings emphasize the value of comprehensive BS evaluations and call for further research with larger sample sizes to validate these observations and enhance our understanding of degenerative spinal changes detected through nuclear imaging.
